# Effects of Binary Mixtures of Inducers (Toluene Analogs) and of Metals on Bioluminescence Induction of a Recombinant Bioreporter Strain

**DOI:** 10.3390/s141018993

**Published:** 2014-10-13

**Authors:** In Chul Kong

**Affiliations:** Department of Environmental Engineering, Yeungnam University, Kyungbuk 712-749, Korea; E-Mail: ickong@ynu.ac.kr; Tel.: +82-53-810-2546; Fax: +82-53-810-4624

**Keywords:** binary mixture, bioluminescence, biomonitoring, inducers, metals, recombinant, toluene analogs

## Abstract

This paper investigated the effects of binary mixtures of bioluminescence inducers (toluene, xylene isomers, *m*-toluate) and of metals (Cu, Cd, As(III), As(V), and Cr) on bioluminescence activity of recombinant (P_m_-*lux*) strain KG1206. Different responses and sensitivities were observed depending on the types and concentrations of mixtures of inducers or metals. In the case of inducer mixtures, antagonistic and synergistic modes of action were observed, whereas metal mixtures showed all three modes of action. Antagonistic mode of action was most common for mixtures of indirect inducers, which showed bioluminescence ranging from 29% to 62% of theoretically expected effects (P(E)). On the other hand, synergistic mode of action was observed for mixtures of direct and indirect inducers, which showed bioluminescence between 141% and 243% of P(E).In the case of binary metal mixtures, bioluminescence activities were ranged from 62% to 75% and 113% to 164% of P(E) for antagonistic and synergistic modes of action, respectively (*p*-values 0.0001–0.038). Therefore, mixture effects could not be generalized since they were dependent on both the types and concentrations of chemicals, suggesting that biomonitoring may constitute a better strategy by investigating types and concentrations of mixture pollutants at contaminated sites.

## 1. Introduction

Petroleum hydrocarbons are some of the most common and important contaminants worldwide [1,2]. A large number of technologies have been developed in order to clean up such sites. However, in some cases, biomonitoring has emerged as one of the most effective remediation strategies, especially where contaminant plumes can be retained within property boundaries in the subsurface and groundwater [3]. This has created a need for a variety of monitoring technologies, ranging from simple and rapid on-site assessments to elaborate laboratory measurements of contaminants [4]. There is also growing interest in techniques based on microbial processes for bioremediation and biomonitoring of contaminated sites, especially those utilizing genetically engineered microorganisms (GEM), in order to enhance degradation of specific pollutants [5]. Numerous microbial processes, including whole cell activity, activities of specific enzymes, intact gene expression, and recombinant gene expression, can be utilized in biomonitoring assays. Such microbially-derived assessment tools are usually simple, rapid, and cost-effective compared to conventional chemical analyses and can be effectively used in the preliminary assessment of contaminated sites.

Advances in understanding of recombinant genes and their regulation have resulted in growing interest in the development of bacterial biosensors, including those applicable to environmental monitoring [6]. For example, bacteria containing recombinant TOL plasmid and the bioluminescence-producing *lux* gene have been investigated in great detail [7]. Genes in the TOL plasmid of *P. putida* encode enzymes that are capable of transforming toluene analogs (components of gasoline) into non-toxic metabolites. The catabolic genes of TOL plasmid are under the control of two regulatory genes, P_s_ and P_r_, which act along with transcriptional activators on two operons, the upper and lower pathways [8]. A biosensor of this type can be genetically engineered by positioning a reporter gene, such as *lacZ* gene encoding β-galactosidase or *lux* genes encoding luciferase and other factors, under the control of a transcriptional activator [6].The *lux* genes of *Vibrio fischeri* (*lux*CDABE) are among the most well known reporter genes, and recombinant strains containing these genes can be utilized for real-time, non-destructive assays based on bacterial production of visible light (bioluminescence). When combined with genes that are activated in the presence of specific chemicals, the *lux* gene sequence of *V. fischeri* acts as an effective bioreporter for environmental monitoring applications. Under appropriate conditions, a recombinant bioreporter gene can produce a signal that is directly correlated to the concentration of a specific pollutant [9]. Such biosensors can generate valuable information about the bioavailability of contaminants in the environment and can serve as useful alternatives to more expensive chemical methods for *in situ* monitoring or on-site analysis.

Environments contaminated with gasoline often contain mixtures, chemicals, metals, and bioluminescence inducers for any particular strain. Therefore, the effects of inducer mixtures containing other chemicals such as metals on bioluminescence production may deviate significantly (antagonistic or synergistic effects)or be similar (additive effect) in relation to theoretically expected effects calculated based on individual chemicals at biomonitoring sites [10].

The majority of biomonitoring studies on pollutants have focused on individual chemicals under controlled conditions [11]. However, in reality, studies focusing only on single chemicals can never provide complete monitoring since the environment is constantly exposed to mixtures of contaminants. In addition, industrialization has resulted in the deposition of complex mixtures of inorganics (heavy metals) and organic pollutants into environments [12]. Especially, mixtures of non-biodegradable pollutants have been found at elevated concentrations in a majority of areas worldwide [13]. Therefore, the interactive effects of chemical mixtures must realistically portray the actual pollution conditions of biomonitoring sites. Previous research has attempted to calculate the joint effects of chemicals using predictive models such as concentration addition and independent action (response addition) [14,15].The theoretically expected effects of binary mixtures on test organisms can be evaluated by using a simple mathematical model based on the theory of probabilities [16] or by the toxic unit (TU) approach [17]. The specific model is clearly based on the mode of action of each pollutant [11]. For example, An [18] reported the effects of a binary metal mixture using the TU approach, which is based on the response addition model. Although the TU approach has limitations, it is useful as a means of comparing the likely relative toxicities induced by heavy metals [17].

The goals of this work were to (1) evaluate the bioluminescence response patterns of KG1206 bioreporter strain exposed to binary mixtures of five inducers (toluene, *m*-toluate, and three xylene isomers); and (2) assess the effects of binary metal mixtures on bioluminescence activity of KG1206 bioreporter strain in the presence of single inducer chemicals.

## 2. Materials and Methods

### 2.1. Strain Characteristics and Culture Conditions

**Figure 1.**
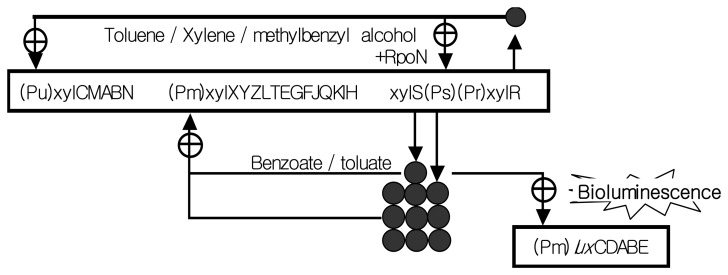
Regulation of TOL catabolic and recombinant *Pm-lux* genes (⊕: positive control; ○: regulatory protein) (adapted from [20]).

The tested recombinant strain *Pseudomonas putida* mt-2 KG1206 (called KG1206)contains the intact TOL plasmid and P_m_-*lux* fusion plasmid, where P_m_ is the promoter of the lower or meta operon (13 genes) of the TOL catabolic plasmid pWW0 and is responsible for producing bioluminescence in the presence of toluene analogs (indirect inducers; toluene, *m*-MBA, xylene isomers) as well as their metabolites (direct inducer; *m*-toluate, and benzoate) (Figure 1) [8,9]. Intermediates of toluene analogs, such as benzoate and *m*-toluate, activate XylS regulatory protein, whereas toluene, *m*-MBA, and isomers of xylene activate XylR regulatory protein. Both XylS and XylR proteins positively control their promoters. XylR controls the upper pathway promoter (P_u_) and induces expression of *xylS* gene, which is responsible for the production of XylS protein as well as regulation of the lower pathway promoter (P_m_). For KG1206 strain, inducer chemicals bind to inactive XylS protein and induce a conformational change, enhancing interaction with the promoter site. Activated XylS protein then binds to the promoter P_m_ to produce bioluminescence from P_m_-*lux* gene in the recombinant strain [7].

KG1206 strain was maintained and stored by using standard procedures [19]. Strains were stored at −70°C until needed, at which time they were grown overnight in Luria-Bertani (LB) medium at 27°C with shaking (130 rpm). A 1:50 dilution in LB medium was made and allowed to grow until the optical density (OD_600_) was approximately 0.6. LB medium consisted of 10 g of tryptone, 5 g of yeast extract, 5 g of NaCl, and 0.5 mL of 2N NaOH per L of broth. LB medium also contained 50 mg/L of kanamycin to prevent bacterial contamination. All reagents, antibiotics, and test chemicals were obtained from Sigma-Aldrich Chemical (St. Louis, MO, USA). Bioluminescence intensity was measured in relative light units (RLU) during incubation using a Turner 20/20 Luminometer (Turner, Sunnyvale, CA USA). The maximum detection limit was 9999 RLU.

### 2.2. Effects of Inducer Mixtures on Bioluminescence Activity

Five inducers (1.0 or 0.5 mM of toluene, *m*-toluate, and three xylenes) were pairwise-mixed and tested (60 combinations in total). Binary mixture effects were determined by measuring bioluminescence after transferring a predetermined amount of bacterial culture (9.9 mL) into serum vials supplemented with appropriate concentrations of binary mixtures of inducer compounds (0.1 mL). The serum vials were sealed with Teflon septa to avoid loss of volatile compounds. The culture was incubated by shaking (130 rpm) at 27°C. During the incubation periods (generally 4 h), bioluminescence production was measured every 30 min. All tests were performed in triplicate.

### 2.3. Effects of Metal Mixtures on Bioluminescence Activity

For the metal mixture test, the toxic unit (TU = concentrations/EC_50_) approach was used, where 1 TU is equal to the EC_50_ concentration of each metal. Based on the EC_50_ for each metal, approximate concentrations of 0.25, 0.5, and 1 EC_50_ of each metal were binary-mixed to assess interactive effects. The experimental design consisted of 90 mixture combinations of CuCl·2H_2_O (97%), CdCl_2_·2H_2_O (98%),NaAsO_2_ (97%),Na_2_HAsO_4_ (99%), and K_2_CrO_4_ (98%). Effects were determined by measuring bioluminescence after transferring a predetermined amount of bacterial culture (9.8 mL) into serum vials supplemented with appropriate concentrations of metal binary mixtures (0.1 mL) in the presence of a single inducer (0.1 mL of 50 mM toluene; final concentration 0.5 mM). The serum vials were sealed with Teflon septa to avoid loss of volatile compounds. The culture was incubated for 4 h by shaking (130 rpm) at 27°C. An average of two measurements (1.0 and 1.5 h) was used to evaluate the results. All tests were performed in triplicate.

### 2.4. Evaluation of Effects of Binary Inducer and Metal Mixtures

To investigate mixture effects, the observed bioluminescence of the binary mixture P(O) was compared with theoretically expected ones P(E), as determined by combining both single test results. In the mixture toxicity test, theoretically expected effects of the binary mixture were evaluated using a simple mathematical model based on the theory of probabilities, which has been used before by several researchers [16]:

(1) $$\text{P}(\text{E})={\text{P}}_{\text{a}}+{\text{P}}_{\text{b}}-({\text{P}}_{\text{a}}{\text{P}}_{\text{b}}/100)$$

where: P_a_: inhibition caused by chemical “a”

P_b_: inhibition caused by chemical “b”

P(E): theoretically expected inhibition

If the difference between P(O) and P(E) was not significant, then the mode of interaction was characterized as additive. The test result was considered to be synergistic or antagonistic only if the observed value was significantly higher or lower, respectively, than the theoretically predicted value at a significance level of *p* < 0.05. Statistical analysis of the experimental groups utilized Student's t test [21]. Concentrations of inducers and metals used in the binary mixtures are shown in Table 1.

## 3. Results and Discussion

Environments contaminated with gasoline often contain mixtures, chemicals, metals, and bioluminescence inducers for any particular strain. Therefore, the effects of inducer mixtures containing other chemicals such as metals on bioluminescence production may deviate significantly (antagonistic or synergistic effects)or be similar (additive effect) in relation to theoretically expected effects calculated based on individual chemicals at biomonitoring sites [10].

### 3.1. Effects of Inducer Mixtures on Bioluminescence Induction

**Figure 2.**
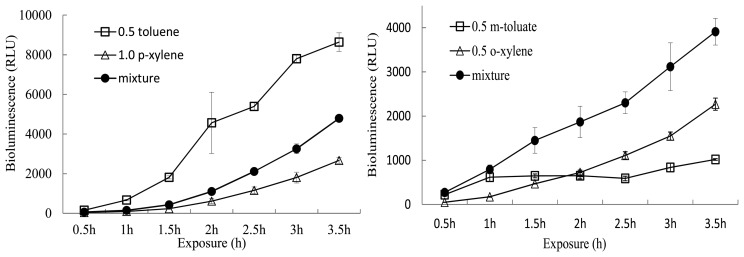
Two representative results of bioluminescence activity in the presence of single inducers and mixtures of binary inducers.

After the initial definitive tests, 1.0 and 0.5 mM each of *m*-toluate, toluene, and *m*-, *o*-, *p*-xylene were used to evaluate the effects of binary mixtures of inducer pollutants on bioluminescence induction. In general, bioluminescence intensities of mixtures were either higher or lower than those of single inducers. Two representative results of the bioluminescence activities of inducer mixtures and single inducers are compared in Figure 2. After exposure for 3.5 h, a mixture of 0.5 mM toluene and 1 mM *p*-xylene produced 4795 RLU, whereas toluene and *p*-xylene as single inducers produced 8640 and 2670 RLU, respectively. In contrast, a mixture of *m*-toluate and *o*-xylene (final 0.5 mM) showed higher bioluminescence (3910 RLU) compared to 0.5 mM *o*-xylene and 0.5 mM *m*-toluate, which showed 2270 and 1918 RLU at 3.5 h, respectively.

**Figure 3.**
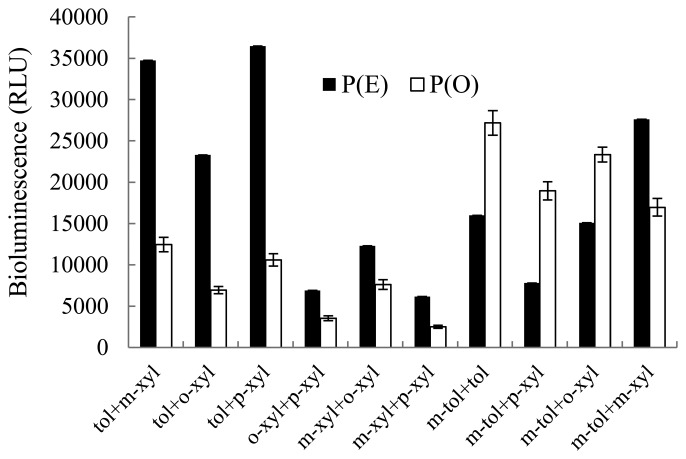
Comparison between observed P(O) and expected P(E) bioluminescence intensities in the presence of binary inducer mixtures. Each bar presents average of P(O) or P(E) bioluminescence intensities of four combinations.

**Figure A1.**
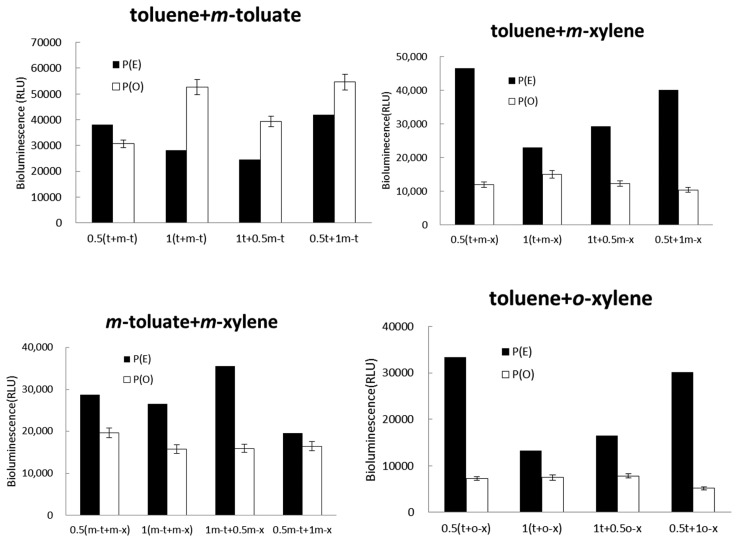

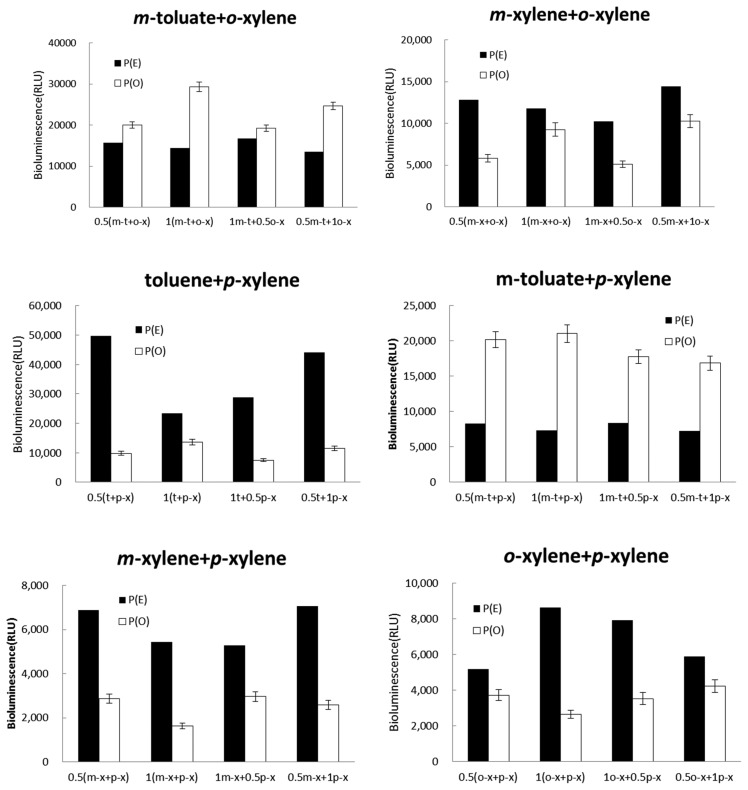
Comparison between P(O) and P(E) bioluminescence intensities in the presence of binary inducer mixtures. *X*-axis “1t + 0.5m-x” represents binary mixtures of toluene and *m*-xylene, and final concentration of each is 1 and 0.5 mM, respectively.

Comparison between expected and observed effects of different combinations of inducer mixtures on bioluminescence induction are shown in Figure 3. Each mixture group consisted of four different binary mixture combinations at two concentrations (Figure A1). Inducer chemicals are known to have similar modes of action. Therefore, expected mixture effects were determined based on the concentration addition model, in which concentrations of all inducer constituents of the binary mixture are added together in order to predict theoretical effects [10]. Observed bioluminescence was in the range of min. 2515 ± 189.8 and max. 25,059 ± 1344.3 RLU, which was 29%–243% of the expected bioluminescence intensity.

For binary inducer mixtures, seven out of 10 mixture groups produced lower bioluminescence (29%–62%) compared to theoretically expected ones P(E). Therefore, the interactive effects of these mixture chemicals were antagonistic. In contrast, three other groups (mixtures of *m*-toluate and toluene, *m*-toluate and *p*-xylene, and *m*-toluate and *o*-xylene) showed higher bioluminescence intensities (141%, 243%, and 155%, respectively) compared to theoretically expected ones (synergistic mode of action), and no additive mode was observed. Based on Student's *t*-test, statistically significant differences were observed between P(E) and P(O) in all groups (*p*-values ranging from 0.0001 to 0.0228).

Synergistic mode was investigated using mixtures of *m*-toluate (direct inducer of P_m_-*lux*) with other inducers and was compared to mixtures of indirect inducers such as toluene and xylene isomers. Mixture responses were dependent on the types of inducer chemicals, such as indirect or direct inducers. Different bioluminescence patterns of inducer mixtures can be attributed to several factors, such as protein conformational changes caused by different inducers, various binding affinities of inducers to the lower promoter site and XylS protein, and many other factors [22]. In general, compounds with low binding affinities for inactive XylS protein result in low bioluminescence at saturating concentrations [22]. Therefore, direct inducers transcribed recombinant P_m_-*lux* gene more efficiently due to strong affinity for XylS protein or binding sites. However, the bioluminescence response was also affected by other factors such as toxic compounds [23], membrane-perturbing chemicals (e.g., organic solvents) [24,25], and other factors (e.g., growth-phase) [26]. These factors should be taken into consideration in order to interpret the effects of mixtures. Especially, the response mechanism of mixtures could be demonstrated by quantifying protein expression levels resulting from different levels of activation.

### 3.2. Effects of Mixture Metals on Bioluminescence Activity in the Presence of Inducer Chemical

**Figure 4.**
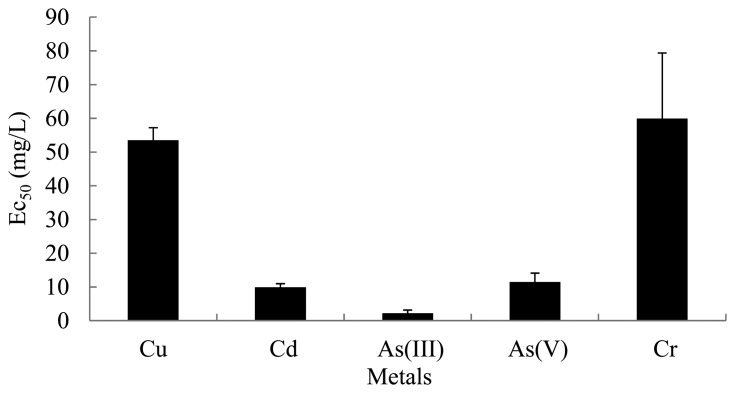
Summary of effects of different metals on bioluminescence induction. Each value represents average EC_50s_ of five inducers with same metal.

**Figure A2.**
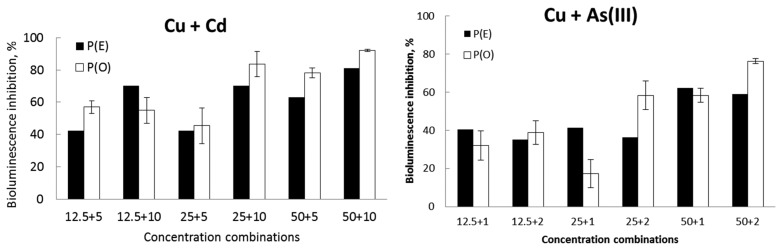

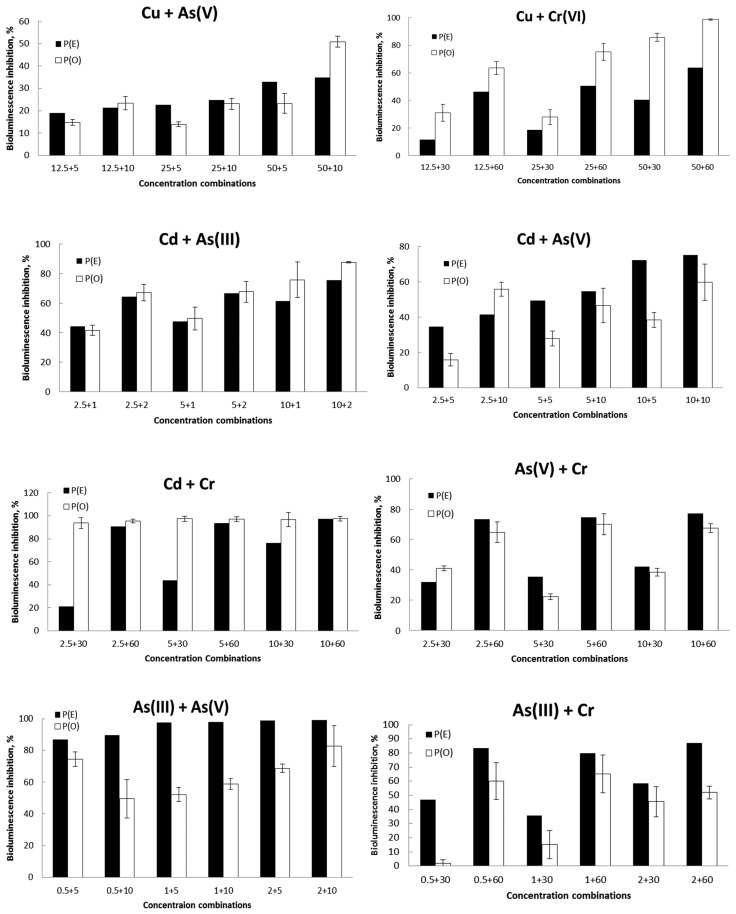
Comparison between P(O) and P(E) bioluminescence intensities in the presence of binary metal mixtures of 0.5 mM toluene. *X*-axis “num1 + num2” represents final concentration (mg/L) of each metal in the binary mixture.

Prior to testing the mixtures, the effects of single metals on bioluminescence activity were determined. Five single metals were exposed to each inducer chemical (toluene, *m*-toluate, and three xylene isomers) (Figure A2). There were no differences between exposure to direct and indirect inducers. However, the same metal had a similar effect (EC_50_) on bioluminescence activity of the strain, regardless of the inducer. Average EC_50_ values of each metal with different inducers are shown in Figure 4. Similar bioluminescence activities were generally observed among the same metal exposed to different inducers. Among the tested metals, arsenite showed the highest inhibitory effects with different inducer chemicals (EC_50_ 1.2–3.8 mg/L) under all tested conditions. On the other hand, copper and chromate showed the lowest effects (EC_50_ 53.5 mg/L and 59.9 mg/L, respectively).Effects of metals were as follows: As(III) 2.2 ± 0.97 mg/L > Cd 9.9 ± 1.07 mg/L, As(V) 11.5 ± 2.60 mg/L ≫ Cu 53.5 ± 3.72 mg/L, Cr 59.9 ± 19.48 mg/L. Toxicities of As(III) and Cd based on bioluminescence activity were much greater than those of Cu and Cr. Chromate (K_2_CrO_4_) toxicity was nearly 25 times lower than that of As(III) in terms of EC_50_. Metals are expected to respond differently to various chemicals depending on type of test organism, test conditions (pH, temperature, physiological state), end points, and so on [27]. Hsieh *et al.* [27] reported the following toxicities by the Microtox method: Cu > Cr > Cd > As. For this reason, a battery of bioassays could be one solution to assess the toxic effects of pollutants [28]. Several mechanisms could explain the lower inhibitory effect of chromate in bacteria: (1) blockage of chromate transport resulting from mutation of sulfate permease protein; (2) chromate-active efflux; or (3) activation of the chromate reduction mechanism. Fulladosa *et al.* [29] reported a low toxicity (high EC_50_ > 2 mM) of chromate with respect to growth of the bioluminescence-producing strain *Vibrio fischeri*. The ability of *V. fischeri* to reduce chromate to chromite may be responsible for the tolerance of the strain to this metal. Although the same metal had a similar effect (EC_50_) on bioluminescence activity, the P_m_-*lux* gene exposed to *o*-xylene was more significantly inhibited (lowest EC_50_ value) by nearly all metals regardless of the inducer. Interestingly, Cu, Cd, and As(V) with *o*-xylene showed the lowest EC values compared to sets with other inducers. Two other metals, As(III) and Cr with *o*-xylene, were not toxic, although their EC_50_ values were within the error range of most toxic metals (data not shown).

**Figure 5.**
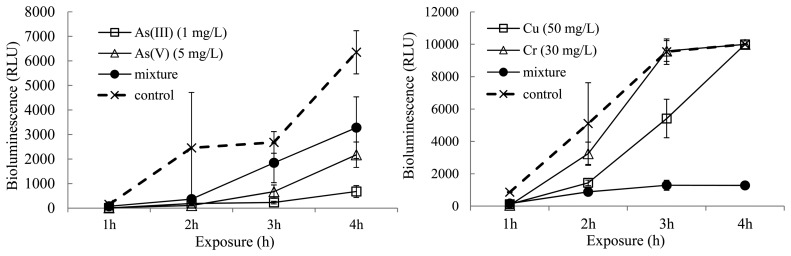
Two representative results of bioluminescence activities of strain KG1206 under different conditions (mixtures of arsenic as well as Cu and Cr in the presence of 0.5 mM toluene).

The interactive effects of metal mixtures were evaluated in the presence of 0.5 mM toluene. Three different concentrations of each metal were used for the binary mixture combinations. Of the tested combinations, two representative results of the bioluminescence activities of strain KG1206 exposed to single or mixture metals in the presence of 0.5 mM toluene are shown in Figure 4. Bioluminescence activities of all sets exposed to metals were lower than that of control (Figure 5).

In general, bioluminescence activities of mixtures were higher or lower than those of single exposures. For example, a mixture of Cu and Cr produced much lower bioluminescence (1287 ± 307.6 RLU) than Cu and Cr separately (5414 ± 1887.1 and 9587 ± 643.6, respectively) after 3 h. In contrast, a mixture of As(III) and As(V) produced higher bioluminescence (3283 ± 1254.2 RLU) than either As(III) or As(V) alone (677 ± 243.5 and 2178 ±519.4, respectively) after 4 h.

**Figure 6.**
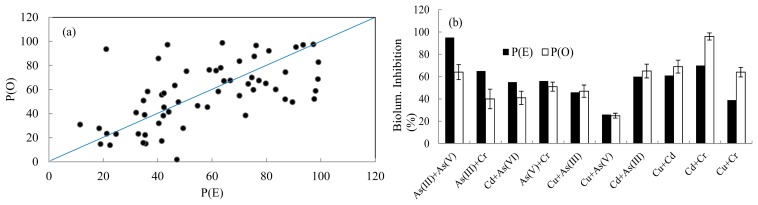
Correlations between theoretically expected and observed bioluminescence activities of KG1206 (mg/L) in the presence of binary mixtures of copper, cadmium, arsenite, arsenate, and chromate. Error bar indicates standard deviation. (**a**) Total combinations (#60); (**b**) average of each set (10 combinations per set).

The observed and expected inhibitory effects by binary mixtures on bioluminescence activity are shown in Figure 6. Among the 60 results for P(O) and P(E), all three different modes of action, including antagonistic (less than additive), synergistic (greater than additive), and additive modes, were wildly distributed. The observed bioluminescence inhibition of the metal mixture was 62%–164% of its expected activity. Based on the average of six combinations of each set, three sets (antagonistic; As(III) + As(V), As(III) + Cr, Cd + As(VI)) showed lower inhibitory effects (62%–75%) on bioluminescence activity compared to theoretically expected ones. Four sets (additive; As (V) + Cr, Cu + As(III), Cu + As(V), Cd + As(III)) produced similar bioluminescence activities (91%–108%) compared to theoretically expected ones. In contrast, three out of 10 sets (synergistic; Cd + Cr, Cu + Cr, Cu + Cd) showed significant inhibitory effects (113%–164%) on bioluminescence activity compared to theoretically expected ones. Based on Student's *t*-test for each mode of action, statistical differences (*p*-value) between P(E) and P(O) were in the range of 0.0001–0.0024 and 0.0001–0.038 for antagonistic and synergistic modes of action, respectively. In contrast, sets for additive modes of action were in the range of 0.0556–0.7593.

Pronounced synergistic effects on bioluminescence inhibition were observed in both binary mixtures of Cr with Cd and Cu (137% and 164% relative inhibition, respectively). Interestingly, Cr alone showed the least effects on bioluminescence activity among all tested metals. Previous studies have reported that metals in mixture scan influence the uptake of other metals [30]. The antagonistic inhibition of bioluminescence could be attributed to the formation of less bio available metal complexes [18].Further, the inhibitory effects of most binary mixtures of Cu were similar to expected values (additive mode of action).This investigation showed synergistic inhibition of bioluminescence induction in binary mixture of Cu and Cd. Norwood *et al.* [10] also reported that pairing of Cu and Cd strictly dominated more than additive interactions. Average values for P(E) and P(O) were 57% ± 24.2% and 56% ± 25.7% (*p* value 0.711), respectively. Similarly, Liu *et al.* [31] reported that mixtures of Cd and As(V) have antagonistic effects on biomass, root, and shoot elongation. These antagonistic effects may be due to reductions in ion activity of the medium when Cd and As(V) are present together, thereby lowering metal uptake [31].

Although the metabolic and transcriptional components of the xyl-gene regulatory system are well known, its architecture is still perplexing [32]. Silva-Rocha [32] revealed that the entire regulatory structure of the TOL system involves the action of a metabolic amplifier motif (MAM). MAM appears to induce simultaneous expression of the upper and lower segments of the inducer catabolic route, which would be difficult to bring about with a standard substrate responsive single promoter.

## 4. Conclusions

The effects of inducer mixtures on bioluminescence activity were difficult to generalize since they were dependent on chemical types and relative concentrations. In general, synergistic and antagonistic modes of action were observed for inducer mixtures, whereas all three modes were observed in the presence of metal mixtures. This result also indicates that biomonitoring using mixtures as opposed to single chemicals is more effective for bio assessment of environmental pollutants [33]. In order to predict interactive joint effects, proper models also must be developed [13]. In addition, further investigating the effects of mixtures at the molecular level on the activity of recombinant bioluminescence strains will clearly reveal additional information about responses [34].

## Figures and Tables

**Table 1.** Concentrations of each chemical in binary mixtures of inducers and metals (mg/L).

Inducers *	**Toluene**	***m*-Toluate**	***o*-Xylene**	***m*-Xylene**	***p*-Xylene**	**60 Binary Mixture Combinations**
Metals (mg/L)	As(III)	As(V)	Cu	Cd	Cr	90 binary mixture combinations
	0.5, 1, 2	2.5, 5, 10	12.5, 25, 50	2.5, 5, 10	30, 60	

* 0.5 mM and 1.0 mM.
